# A Case of Symptomatic Supratherapeutic International Normalized Ratio on Rivaroxaban

**DOI:** 10.7759/cureus.40282

**Published:** 2023-06-12

**Authors:** Angela Khidhir, Farhan Azad, Matthew Gravina

**Affiliations:** 1 Department of Medicine, University at Buffalo Jacobs School of Medicine and Biomedical Sciences, Buffalo, USA; 2 Internal Medicine, University at Buffalo, Buffalo, USA; 3 Hematology and Medical Oncology, University at Buffalo, Buffalo, USA

**Keywords:** direct oral anti-coagulants, international normalized ratio, high inr, supra-therapeutic inr, rivaroxaban

## Abstract

Rivaroxaban is a direct oral anticoagulant that works by inhibiting factor Xa. Direct anticoagulants have largely replaced direct vitamin K inhibitors (VKAs) due to the decreased risk of major hemorrhages and the lack of need for regular monitoring and dose adjustments. However, there have been multiple reports of elevated international normalized ratio (INR) and bleeding incidents in patients on rivaroxaban, which brings into question the potential need for monitoring. We report a case of an INR of 4.8 in a rivaroxaban-naïve patient who presented with gastrointestinal bleeding and a significant drop in hemoglobin four days after starting rivaroxaban. We present possible pharmacologic explanations. We propose the idea that specific subgroups of patients may be at risk for true INR elevations and may benefit from routine monitoring of their INR while on rivaroxaban.

## Introduction

Direct vitamin K inhibitors (VKAs) and aspirin have traditionally demonstrated excellent efficacy in the prevention of stroke and thromboembolism. However, there are many disadvantages associated with them, including the risk of major hemorrhages and difficulty managing them due to many drug-drug and drug-food interactions, and non-predictable pharmacokinetic profiles requiring regular monitoring and dose adjustment [1,2]. Direct oral anticoagulant drugs (DOACs) were developed to counter the disadvantages that are associated with direct VKAs. They have several approved indications including reducing the risk of stroke and systemic embolism of non-valvular atrial fibrillation, treatment of recurrent deep venous thrombosis and pulmonary embolism, and venous thromboembolism [3]. Rivaroxaban is a DOAC shown to significantly reduce the incidence of acute limb ischemia, major amputation, myocardial infarction, ischemic stroke, or death from cardiovascular causes in patients with peripheral vascular disease who have undergone lower-extremity revascularization at a dose of 2.5 milligrams when combined with aspirin [4].

Prothrombin time (PT) is the most commonly used laboratory value to test for coagulation [5]. PT value can vary between different laboratories based on the sensitivities of available thromboplastic agents to the reduction in coagulation factors. The international normalized ratio (INR) is used instead to correct for this variability, which makes it utilizable regardless of the reagent used. It is calculated by using the manufacturer’s international sensitivity index (ISI), which is the ratio of the responsiveness of a particular thromboplastic agent to the reduction of coagulation factors relative to the primary World Health Organization (WHO) international reference preparations [6].

Direct VKAs require routine monitoring of INR due to their variable pharmacokinetic profile. The development of DOACs, including rivaroxaban, has negated the need to do so. However, there have been multiple reports of elevated INR while on rivaroxaban and incidents of bleeding, which raises the question of the potential need for monitoring. We report a case of an INR of 4.8 in a patient with no liver or kidney function abnormalities who presented with a gastrointestinal bleed and a drop of 5 g/dL in hemoglobin four days after starting rivaroxaban.

## Case presentation

A 90-year-old Caucasian male with a past medical history of hypertension, hyperlipidemia, coronary artery disease with percutaneous coronary intervention (PCI), peripheral artery disease with left femoral endarterectomy, and superficial femoral artery angioplasty, right femoral to popliteal (PT) polytetrafluoroethylene (PTFE) grafts with vein patch presented with shortness of breath four days following an open endarterectomy of the right femoral PT bypass with stenting. The patient had been discharged on rivaroxaban 15 milligrams twice daily (BID) and aspirin 81 milligrams once daily (QD). He had loose burgundy stools the day before he presented.

Laboratory values on re-admission showed a hemoglobin (Hgb) of 7.0 g/dL (reference range: 12.0-16.0 g/dL), which was a drop from a Hgb of 13.1 g/dL on the day of his discharge four days prior, and an INR of 4.8 (reference range: 0.00-3.50). Computed tomography did not reveal any hematoma at the surgical site. Rivaroxaban was held and he was transfused multiple units of packed red blood cells as the transfusion cutoff for this patient was at Hgb of 8.0 g/dL. Esophagogastroduodenoscopy (EGD) and colonoscopy did not reveal the source of bleeding. A multi-disciplinary approach was taken to decide whether rivaroxaban should be restarted. The gastroenterology team advised that there was a high probability of rebleeding should the medication be restarted. The patient was ultimately discharged on dual antiplatelet therapy which he had been taking prior to the open endarterectomy procedure.

The patient had never been on a DOAC before. He did not have any hepatic or renal disease. Liver function tests were within normal limits. Creatinine and estimated glomerular filtration rate (GFR) were within range. His other medications included acetaminophen 650 milligrams every six hours as needed, amlodipine 5 milligrams QD, atorvastatin 80 milligrams QD, carvedilol 3.25 milligrams BID, furosemide 40 milligrams QD, lisinopril 10 milligrams QD, metformin 500 milligrams BID, pregabalin 350 milligrams QD, fish oil 1000 milligrams BID, and a multivitamin. The patient did not ingest any grapefruit and denied taking any other over-the-counter medications or herbal supplements.

## Discussion

The literature has documented false elevated INRs on anticoagulation [7]. However, the high INR was associated with a gastrointestinal bleed in this patient. Therefore, this is considered a true elevation, and the etiology of that should be investigated.

Rivaroxaban has a high volume of distribution of 50 L and a prolonged half-life in older adults. Consequently, the patient’s low body mass index of 20 kg/m^2^ (reference range: 18.5-24.9 kg/m^2^) and advanced age could have contributed to increased levels of the drug, leading to elevated INR and risk of bleeding. The patient’s race could also have contributed. In a study comparing rivaroxaban concentrations between Singaporeans and Caucasians, Caucasians were found to have higher steady-state trough and peak concentrations [8].

About one-third of rivaroxaban is hepatically cleared through oxidation by cytochrome P450 3A4 (CYP3A4), cytochrome P450 2J2 (CYP2J2), or hydrolyzed to inactive metabolites. Although non-dihydropyridine calcium channel blockers are known to be CYP3A4 inhibitors, dihydropyridines such as amlodipine, which the patient was taking, were found to be only weakly inhibitory of the enzyme [9]. The patient was on aspirin. However, aspirin therapy may decrease, not increase, the INR. Although angiotensin receptor blockers (ARBs) may be associated with lower INRs, as previously mentioned, the patient was on lisinopril. Angiotensin-converting enzyme inhibitors (ACEIs) were found to downregulate tissue factor synthesis, which may inhibit coagulation. However, this has yet to be thoroughly studied [10].

The literature review did not reveal evidence that fish oil could inhibit clearance mechanisms. However, the high volume of distribution of rivaroxaban indicates that it is relatively lipophilic. Given that fish oil can form emulsions in the blood, and the patient was taking it BID along with rivaroxaban, it is possible that it formed an emulsion with the drug, thus prolonging its half-life [11,12].

The patient was not taking any p-glycoprotein (p-gp) and ATP-binding cassette subfamily G member 2 (ABCG2) inhibitors. However, he could have a polymorphism in the p-gp gene or the ABCG2 gene, seen in 2-10.5% of Caucasians, which would influence the drug’s metabolism [13,14]. The patient could also have a polymorphism in the CYP2J2 gene, which is thought to affect about 2% of the general population [15].

## Conclusions

In conclusion, we presented a case of a true elevation of INR in a rivaroxaban-naïve patient that was associated with a drop in Hgb. This case suggests the possibility of increased drug levels or activity in subgroups of patients without pre-existing liver or kidney function abnormalities. The next step is to conduct studies to identify these groups of patients and understand the pharmacokinetic and pharmacodynamic reasons influencing their response to rivaroxaban. These patients may benefit from routinely monitoring their INR, drug level, or factor Xa activity to prevent bleeding incidents.
